# The Focused Neurosurgical Examination During Telehealth Visits: Guidelines During the COVID-19 Pandemic and Beyond

**DOI:** 10.7759/cureus.13503

**Published:** 2021-02-23

**Authors:** Gregory Basil, Evan Luther, Joshua D Burks, Vaidya Govindarajan, Timur Urakov, Ricardo J Komotar, Michael Y Wang, Allan D Levi

**Affiliations:** 1 Neurological Surgery, University of Miami Miller School of Medicine, Miami, USA

**Keywords:** telemedicine, telehealth, neurosurgery, neurologic exam, covid-19, coronavirus

## Abstract

Objective: To provide guidelines to healthcare workers for performing a focused neurological examination via telemedicine during the coronavirus disease-2019 (COVID-2019) pandemic.

Methods: We reviewed our department’s outpatient clinic visits after the implementation of a telemedicine protocol in response to the COVID-19 crisis. Crossover rates from telehealth to in-person visits were evaluated and guidelines for performing a telemedicine neurological exam were created based on the consensus of 16 neurosurgical attending providers over a four-month period.

Results: From March 23, 2020 to July 20, 2020, some 2157 telehealth visits were performed in our department. Some 26 were converted to in-person visits by the provider request with the most cited reason for conversion being the need for a more detailed patient evaluation. Based on these experiences, we created a graphical tutorial to address the key components of the neurological exam with adaptations specific to the telehealth visit.

Conclusions: In response to the global coronavirus pandemic, telemedicine has become an integral part of neurosurgeons’ daily practice. Telemedicine failures remain low but primarily occur due to a need for more comprehensive evaluations. We provide guidelines for the neurosurgical exam during telehealth visits in an effort to assuage some of these issues.

## Introduction

In modern medicine, telehealth has often been focused on access to care in remote, resource-limited areas [1-3]. However, the global coronavirus disease 2019 (COVID-19) pandemic has shifted traditional practice patterns with many now considering telemedicine an integral part of daily outpatient care [4-9]. Despite success in different subspecialties, incorporation into neurosurgical practice has remained slow, in part, because the neurological exam remains paramount for treatment decisions [10]. Furthermore, there is a paucity of literature investigating the reliability and nuances of adapting the neurological exam to telemedicine for the neurosurgical patient [11-13]. It is the intention of this study to demonstrate the feasibility of the focused telemedicine neurosurgical exam and provide guidance to healthcare providers entrusted to perform neurological exams remotely. 

## Materials and methods

A retrospective review of billing records for outpatient telemedicine clinic visits performed by neurosurgical attending physicians from March 23, 2020 to July 20, 2020 was performed. Telemedicine visits were defined by the current procedural terminology (CPT) codes G2010/2012 or the usage of CPT modifier 95. A telemedicine failure was defined by a provider requested in-person clinic visit following the telemedicine visit. These failures and the reason for telemedicine inadequacy were tabulated and reported as percentages. Using the experience of the 16 participating neurosurgical attending providers and these findings, consensus guidelines for performing a focused telemedicine neurologic exam were constructed.

## Results

Telemedicine failures and inadequacies

Some 2157 telehealth visits occurred from March 23, 2020 to July 20, 2020. Some 26 telemedicine failures were identified. Some 21 were spine patients and five were cranial. Some 19 (73.1%) occurred because a more detailed evaluation was needed and 14 of those encounters had specific information regarding what circumstances necessitated an in-person visit. Some 50% cited inability to remotely evaluate radiographic images as the reason for conversion to an in-person visit. The other 50% were due to the need for a better physical examination yet only four required more focused neurological evaluations and only one was to better evaluate a motor deficit. Table 1 displays these results. Based on these experiences, we created a graphical tutorial to address the key components of the neurological exam with adaptations specific to the telehealth visit in an effort to mitigate these conversions.

**Table 1:** Cited reasons for provider-requested telemedicine crossovers. *Patient evaluated for cutaneous stigmata of neurofibromatosis

Reason	Frequency (#)	Percent total (%)
Need for a more detailed evaluation	19	73.1
Inability to remotely review imaging	7	50.0
Wound evaluation	2	14.3
Potential seizure activity	2	14.3
Gait evaluation	1	7.1
Motor evaluation	1	7.1
Other evaluation*	1	7.1
Surgical discussion	5	19.2
Infection	1	3.8
Need for interpreter	1	3.8

The focused neurosurgical telehealth examination

It is important to first recognize that most telehealth visits are performed with patients alone in their home. Although this may initially feel prohibitive, we have found that most medical tools seen in the clinic have household analogs with the exception of the reflex hammer. Keeping these limitations in mind, we have developed the following guidelines for performing remote examinations on neurosurgical patients.

Mental Status

The mental status exam is generally used to assess overall alertness and orientation. Since we are typically not evaluating subtle neurocognitive deficits in neurosurgery, this focused examination need not encompass the exhaustive components seen in a full mental status exam. Instead, a practitioner can simply perform the classic “Mini-Mental Status” questionnaire -- the utility of which is well documented in the literature [14]. For the portions of this exam that require a patient to write down responses, the patient should hold their answers up to the camera for the practitioner to view.

Motor Strength

First, we must accept that a motor exam performed remotely will likely be biased by the patient’s subjectivity. In an effort to counteract this, we suggest modifying the traditional strength grading scale. The proposed modifications are as follows: no muscle movement, some movement but not anti-gravity, anti-gravity, and more than anti-gravity.

**Figure 1:**
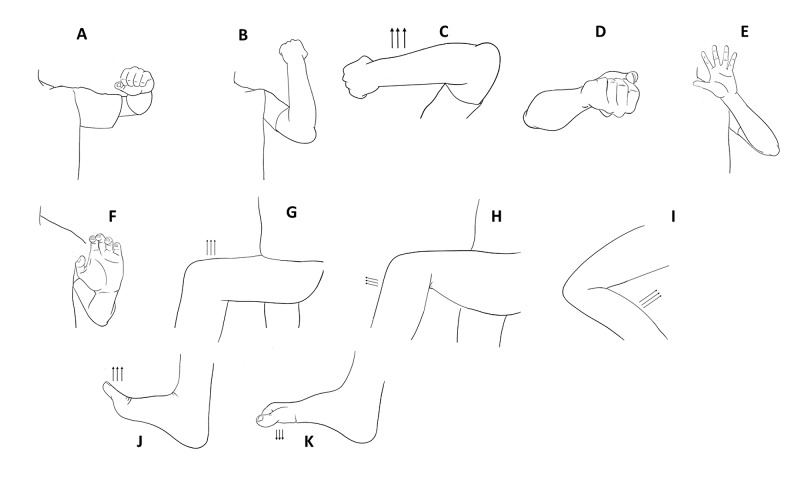
Positions for strength tests to evaluate antigravity movement. (A) Deltoids, (B) biceps, (C) triceps, (D) finger flexion, (E) hand intrinsics/finger extension, (F) wrist extension, (G) iliopsoas, (H) quadriceps, (I) knee flexors, (J) ankle dorsiflexion, and (K) ankle plantar flexion.

To begin the strength exam, we propose having the patient mimic a number of actions. These movements should focus on isolating each muscle group and are displayed in Figure 1. To test whether the patient’s strength is greater than anti-gravity in the upper extremities, we recommend asking the patients to find a weighted object in their home and to repeat the movements while holding the object. For the lower extremity exam, determining strength is slightly more challenging. For the iliopsoas, a simple maneuver would be to have the patient open a large book (if available), and lay it opened over their thigh. The patient should then be asked to bring their knee upwards towards their chest (now weighted slightly by the book). If they are able to do so, they have earned the designation of “more than anti-gravity”. Both quadricep and knee flexor strength can be tested by having a patient squat and then stand. The ability to stand and squat will earn them the designation of “more than anti-gravity”. In terms of tibialis anterior and extensor hallicus longus strength, the patient should be asked to point their toes upwards and take several steps forward on their heels. If they are able to perform these tasks, they earn the strength grade of “more than anti-gravity”. Likewise, for gastrocnemius testing a patient can be asked to walk on their “tip toes”, a practice commonly performed in the office already. Of note, the patient should be asked to do this maneuver next to a wall to prevent any falls should they be unstable or unable to perform the maneuver.

Sensation

**Figure 2:**
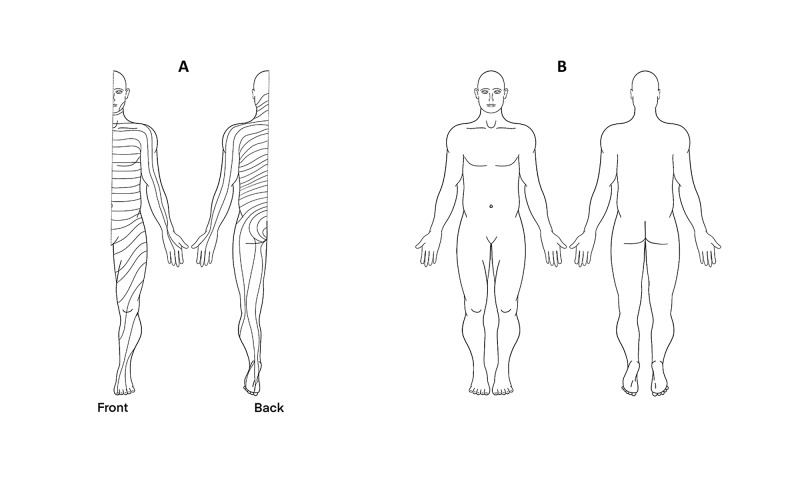
Dermatomal map (A) and corresponding blank diagram (B) to demonstrate distributions of sensory symptoms.

While the standard neurological exam calls for a detailed evaluation of various sensations, this type of granularity is not practical via telemedicine. Instead, we must rely on patient reported symptoms. As such, we recommend allowing the patient to demonstrate on an unlabeled dermatomal map the dermatome most closely resembling their symptoms (Figure 2A). This can be accomplished via screen sharing, with the practitioner slowly moving the mouse from dermatome to dermatome until the patient identifies an abnormal area. By omitting labels from the dermatomal map, we prevent the patient from unconsciously biasing their sensory diagram based on their radiological findings. If they are unable to localize their symptoms on a dermatomal map, they can describe the location of their symptoms on a blank diagram (Figure 2B). In such a manner, the examiner can distinguish dermatomal sensory deficits from other patterns of sensory loss.

Reflexes

**Figure 3:**
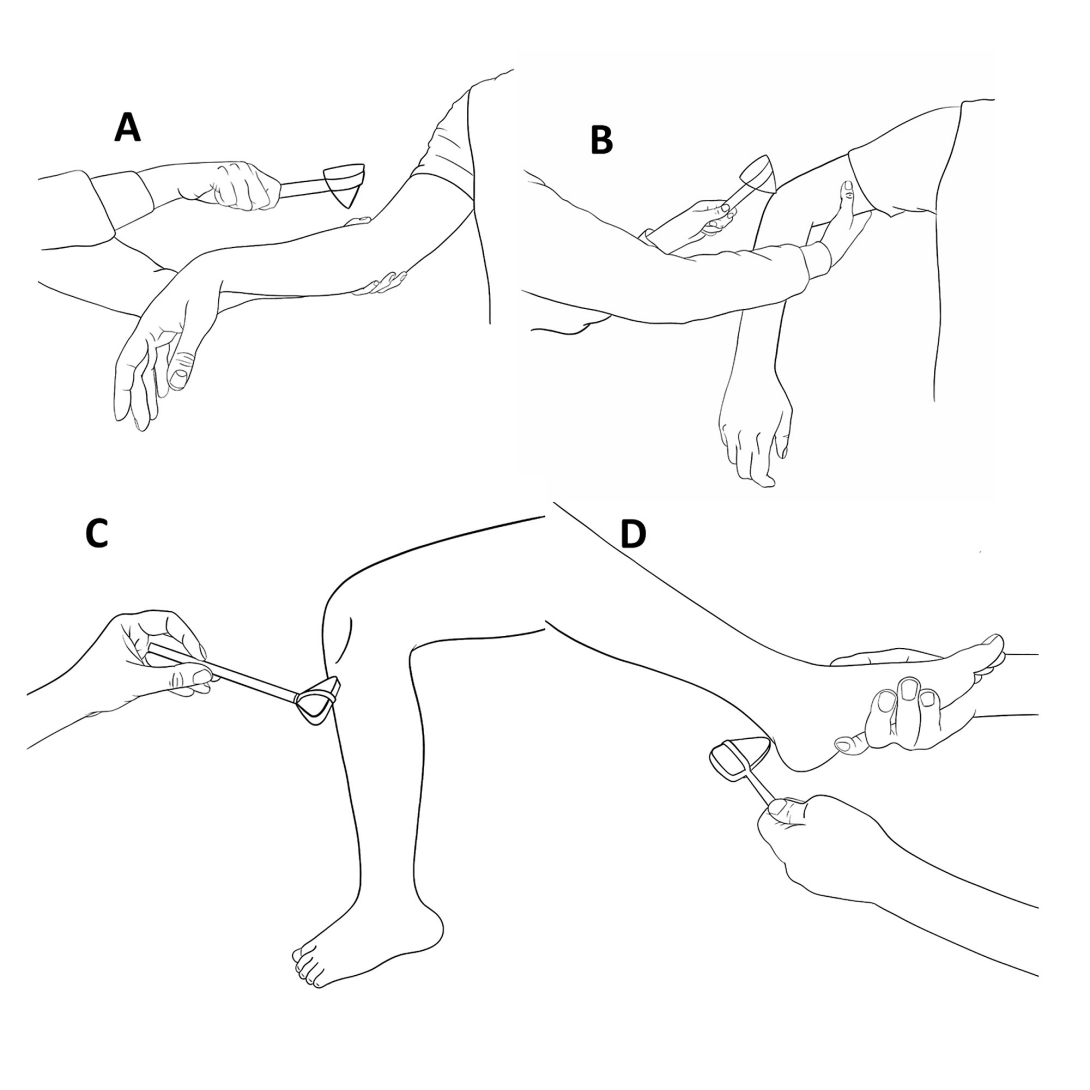
Recommended techniques for eliciting a biceps (A) triceps (B), patellar (C), and Achilles (D) reflex.

A thorough reflex exam can be very difficult to perform via a telemedicine encounter especially given that the closest analog to a reflex hammer in the home are the tips of the patients’ fingers. Thus, while reflexes can be self-elicited, the practical performance of such an exam is challenging for most patients. As such, we recommend that if a family member is present, that they attempt to perform the exam on the patient under direct observation of the practitioner. Given that some reflexes are more easily elicited than others, we recommend simplifying the reflex exam to the following reflexes: biceps, triceps, patellar, and Achilles. Figure 3 depicts the techniques used to elicit these reflexes with a standard reflex hammer.

If no family member is present, we recommend further simplifying the exam so that the patient may perform it on themselves. Asking them to attempt to elicit a biceps and patellar reflex at minimum provides gross information regarding reflexes of the upper and lower extremities. However, if the patient is experiencing difficulty with this portion of the exam we recommend deferring it.

**Figure 4:**
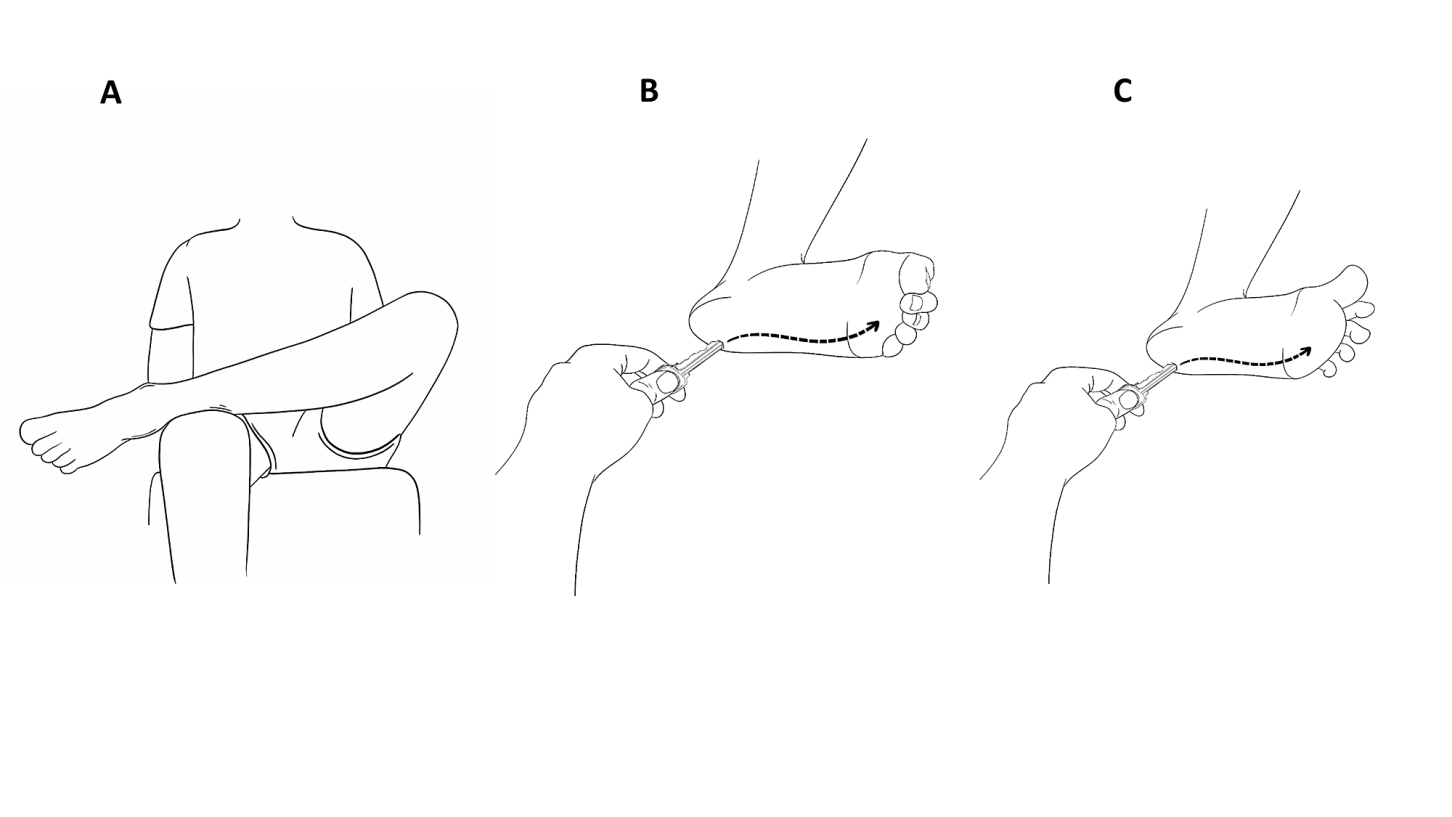
(A) Positioning of a self-administered Babinski test with (B) a normal plantar reflex and (C) a Babinski sign.

While there are a number of tests commonly employed to evaluate for cervical myelopathy, the Babinski sign has been demonstrated to have a high positive likelihood ratio and post-test probability [15]. Fortunately, it is also relatively straightforward to perform and there is evidence that a Babinski sign can be easily self-induced with self-elicitation reducing the likelihood of mistaking it as withdrawing from pain [16]. Considering this fact, patients should be encouraged to perform the Babinski in the home setting in view of the camera. To perform this maneuver, they should be asked to cross one leg over the other, so that the foot being tested is resting on the contralateral thigh (Figure 4A). They should then be instructed to obtain a pointed instrument, such as a key, to perform this exam. Next, they will perform a single sweeping motion along the lateral aspect of the sole of the foot, beginning posteriorly at the calcaneus, and sweeping anteriorly towards the balls of the feet (Figure 4B,C). The clinician should have the camera trained to the feet during this maneuver to determine the presence or absence of a Babinski sign.

Unfortunately, self-performance of a Hoffman’s exam is more challenging and not well documented in the literature. We would therefore recommend differing this portion of the examination.

Cranial Nerve (CN) Examination

CN I: Olfactory nerve

Loss of smell can certainly be representative of neurosurgical pathology, such as an olfactory groove meningioma. Frequently symptoms of anosmia or changes in olfaction are noticed by the patient and thus assessed during the history. Considering this fact, there seems little value in attempting to adapt a novel technique to this component of the exam. Instead, we recommend performing a thorough history to assess if the patient has experienced any changes in smell, and if so, the time course of these changes.

CN II: Optic nerve

Many patients visit the neurosurgical clinic with visual complaints who have not yet had a detailed optic nerve exam. Therefore, we recommend using the full-size Snellen eye chart, which can be displayed via screen sharing, to assess visual acuity one eye at a time. The patient should be asked to cover one eye with their hand during the exam and read-off from the chart moving downwards in typical fashion. It is critical to ensure that the patient is standing 20 feet from the chart during this exam and that the chart is displayed true to size on the patient’s screen. A rudimentary visual field examination can also be performed by asking the patient to cover one eye while holding the other arm as far laterally as possible. The important point here is to make sure that the clinician can see both the patient’s face and hand during the entirety of the exam. Asking them to wiggle their fingers, they should bring their hand closer to their face until they can visibly identify the fingers. This should be repeated in all four quadrants of both eyes. In this way, the clinician can gauge whether there are any gross visual field deficits.

**Figure 5:**
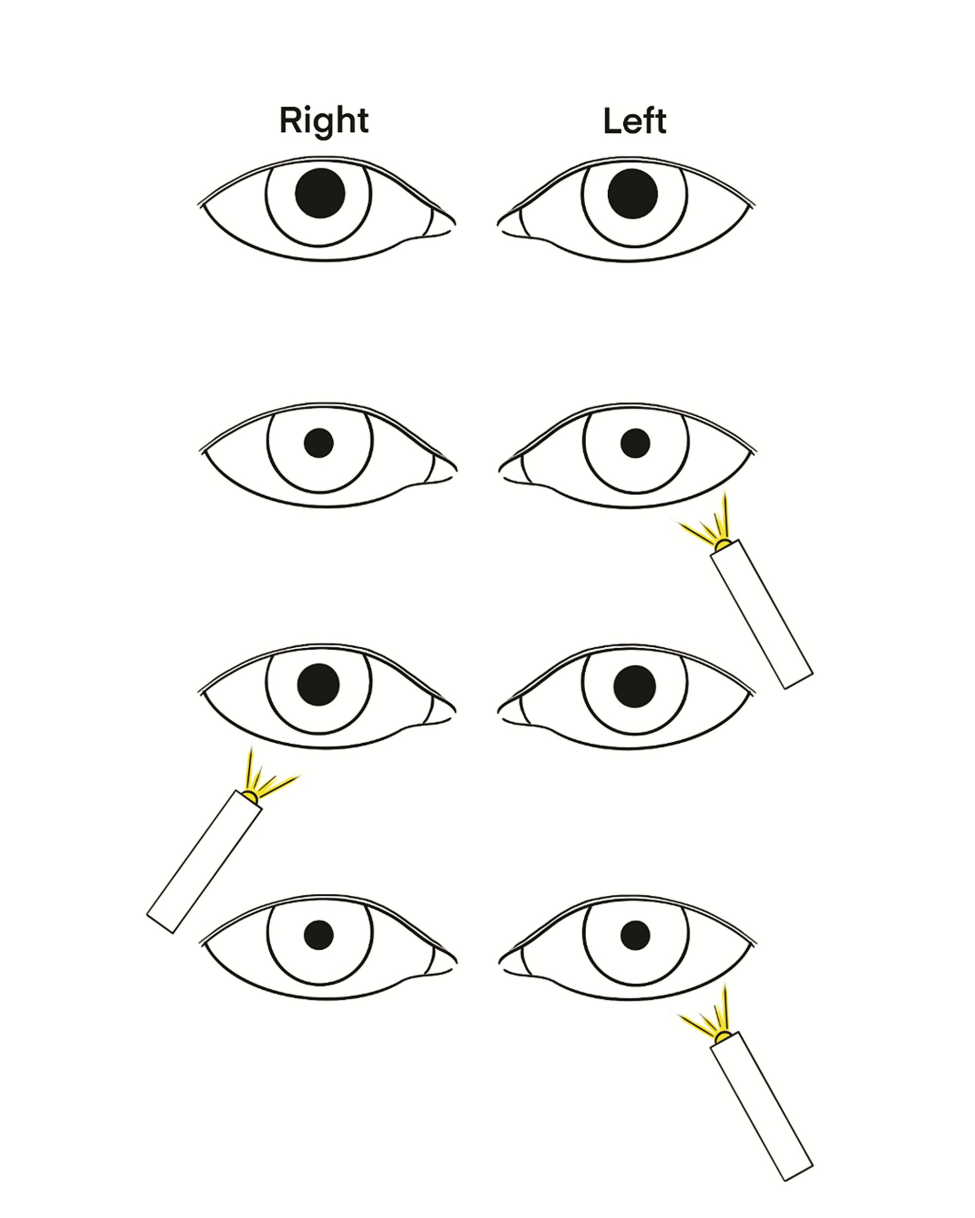
Detection of an afferent pupillary defect in the right eye using a swinging light test.

The pupillary reflex can also be performed remotely to determine optic nerve functionality. The clinician should ask the patient to direct a flashlight into their pupils while looking at their camera. If the pupil reacts, this would be considered a positive response for both CN II and CN III. If the pupil does not react and is large, the practitioner should ask the patient to check the contralateral pupil. If this pupil does react, they should be asked to perform the swinging light test on the first, nonreactive pupil to determine whether there is an afferent or efferent defect (Figure 5). If further examination (such as a fundoscopic exam) is needed, then the patient may require an in-person evaluation.

CN III:

Motor testing of CN III can be done by asking the patient to follow your finger as you move it from side to side and up and down. Any deficits should be easily observed and will also satisfy the assessment of CN IV and CN VI. It is, however, helpful to ask the patient to sit as closely as possible to the camera to evaluate for any subtle dysfunction and to ask the patient if they have any diplopia during eye movement.

The parasympathetic component of CNIII can again be assessed by having the patient direct a flashlight into their pupils while looking at the camera. Again, in the setting of a nonreactive pupil, the swinging light test can be useful in determining whether this defect is afferent or efferent with an efferent defect suggesting CNIII parasympathetic dysfunction. The clinician should also qualitatively assess for ptosis, as its presence would also be suggestive of CNIII dysfunction.

CN IV:

The assessment of CN IV is slightly more complicated than CN III, however, it does not require any significant adaptation to a telemedicine encounter.

**Figure 6:**
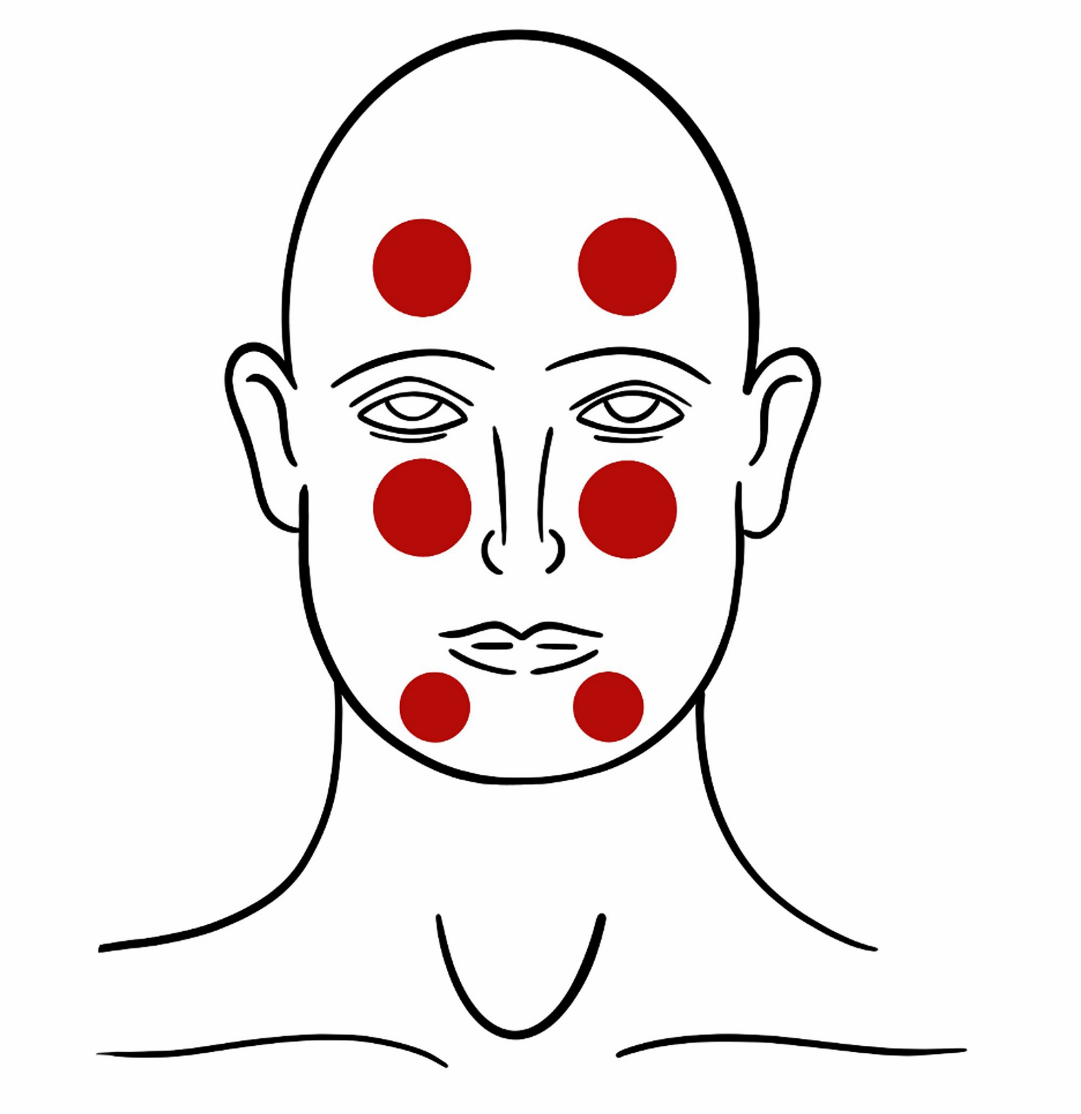
Diagram demonstrating distributions of sensory symptoms to evaluate for trigeminal nerve dysfunction.

CN V:

While CN V has both a motor and a sensory component, practically speaking the motor component cannot be evaluated remotely. Therefore, our focus will be on the evaluation of the sensory component. Similar to the dermatomal sensory exam of the body, the clinician should provide the patient with a map of the face demonstrating three zones and allow the patient to describe their symptomatology based on this diagram (Figure 6).

CN VI:

As is the case with all the pure motor cranial nerves, CN VI can easily be assessed remotely in traditional fashion.

CN VII:

Examination of the motor component of CN VII is of paramount importance. This portion of the exam also requires no adaptation for a telemedicine visit. A facial nerve exam should be therefore performed in the usual fashion and scored using the House-Brackman grading system [17].

CN VIII:

The vestibulocochlear nerve provides an interesting challenge to a telemedicine encounter. While tests such as the Rinne and Weber are commonly used to distinguish conductive hearing loss from sensorineural hearing loss, they are not practical for a telemedicine encounter (in the absence of another advanced practitioner at the bedside). There are, however, gross means with which cochlear nerve function can be tested.

In the outpatient setting, the use of computer-based audiometry shows significant promise in addressing the telemedicine needs of a neurosurgical practice [18]. This allows for detailed audiological examination without a face-to-face encounter, using only a computer with a high-quality sound-card, a tele-audiometric program, and a calibration module [18]. However, although there is evidence in favor of remote audiometry in telemedicine, this requires a significant investment in both equipment and training [18-19].

Alternatively, other measures can be taken to examine gross hearing if a family member is present such as the “whispered voice test” [20]. This test can be performed by asking the family member to occlude one ear while whispering three random letters or numbers into the contralateral ear [20]. It is also recommended that prior to performing the actual test, a trial is performed whereby one ear is occluded as described above, while the number “99” is said loudly into the contralateral ear [20]. If the patient is unable to repeat any of the three numbers, the test should be repeated in the ipsilateral ear. A patient is considered to have “passed” the test if three out of six numbers are repeated correctly [20]. If a family member is not available then this portion of the exam will have to be omitted.

CN IX:

There is no reliable and safe method to effectively evaluate glossopharyngeal nerve function in via outpatient telemedicine. Therefore, glossopharyngeal function should be evaluated by taking a detailed history, with specific questions regarding swallowing and taste/sensation on the posterior 1/3 of the tongue.

CN X:

Perhaps the easiest means of testing CN X function is by having the patient open their mouth wide and say “Ah”. They should do this close to the camera so that any potential uvular deviation can be evaluated. While there are other tests which could be performed to test CN X function, this test is easily performed, and does not require the use of an assistant or any advanced maneuvers.

CN XI:

As CN XI is a pure motor nerve, it lends itself well to a virtual assessment. The patient can simply be asked to shrug their shoulders and turn their head to either side. As these commands are given, the patient should closely be observed to ensure symmetric shoulder elevation and activation of the sternocleidomastoid muscles.

CN XII:

CN XII is also a pure motor nerve and can be assessed virtually by asking the patient to stick their tongue straight out while looking at the camera. A deviation to any side indicates a possible CN XII neuropathy on the side ipsilateral to tongue deviation.

Gait

The gait exam via telemedicine is no different than that performed in-person except for the likely absence of an assistant. For this reason, if a family member is not present, patients should first be questioned about their ability to walk safely without assistance. If they feel safe walking independently, then they should proceed by walking normally across the room within site of the camera. This should be repeated using heel, toe, and tandem walking and should be done next to a bed or wall to assure they have something to stabilize themselves if necessary. If they report that they are unable to walk unassisted, the patients should be asked to walk with the device they typically use. The heel, toe, and tandem walk should also be avoided in these patients.

Cerebellar Function

Cerebellar testing does not require any significant adaptation for telemedicine and should be performed in the usual fashion.

Kernig and Brudzinski Tests

Given the significant amount of data questioning the utility of these tests in diagnosing acute meningitis, we recommend an in-person evaluation and lumbar puncture (LP) for any patient where there is a high clinical suspicion of meningitis rather than attempting to perform these tests remotely [21-22].

Straight Leg Raise (SLR) Test

**Figure 7:**
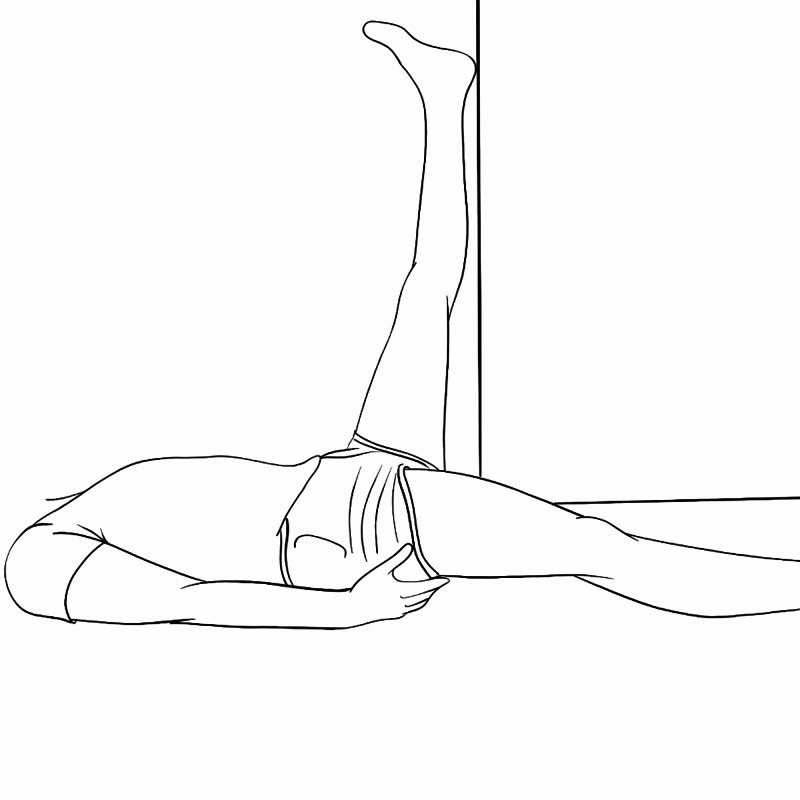
Self-administered SLR test. SLR, straight leg raise

A straight leg test may need to be performed for patients presenting with new-onset radiculopathy. In order to adapt the SLR test to telemedicine, we recommend patients find a corner wall and lift the affected leg up so that the heel rests against the wall while the other leg remains extended (Figure 7). If radicular pain is provoked with this maneuver it can be considered a positive sign. Of note, this procedure may not be possible in the elderly given the strength and flexibility required. In this population, this component of the exam should be deferred.

Fortin Finger Test

The diagnosis of sacroiliac (SI) joint dysfunction is one which often requires multiple clinical tests and is often informed significantly by the clinical history. There are multiple tests which can be performed to help strengthen this diagnosis including the Patrick test, Gaenslen’s test, and the Fortin finger test [23-24]. However, considering the limitations of the telemedicine encounter, the test for SI dysfunction which seems best suited for these interactions is the Fortin finger test. This entails asking a patient to use the tip of their finger to point to the spot on their back where the pain is worst. If this point is within approximately 1 cm of the SI joint, this is considered a positive test [23-24]. While the Patrick test is also commonly employed, this test requires significantly more maneuvering, and is therefore less practical for a virtual encounter. Please note, however, that previous studies have discussed the need for multiple tests in order to appropriately diagnosis SI joint dysfunction. It is therefore, quite possible that the telemedicine encounter must serve as an initial screening for SI dysfunction, but further testing (such as injections into the SI Joint), and possibly face-to-face examination may be required [25].

## Discussion

Due to unprecedented challenges resulting from the global COVID-19 pandemic, neurosurgeons no longer have the luxury of face-to-face interactions and must provide innovative solutions to continue providing care to patients. The intention of this study was to provide an in-depth discussion of the technical means and nuances of adapting the neurological exam to telemedicine in order to provide guidance to healthcare workers during telehealth neurosurgical examinations. Although it is clear that a comprehensive neurological examination cannot be completed remotely, our results show that provider-initiated conversions to in-person visits for more detailed evaluations occurred in only 0.8% of all telehealth visits. This suggests that despite the limitations of the telehealth examination, in most cases the neurosurgical providers gained enough information during the telemedicine visit to create an informed treatment plan. While the methods described in this paper require external validation, they do provide an important blueprint for a way forward. Furthermore, while some may view telemedicine as a temporary “stop-gap” during the global pandemic, it is more likely that a permanent paradigm shift in medicine will occur with many adopting a hybrid clinical practice utilizing both telemedicine and traditional in-person visits.

Our results demonstrate that of the small number of patients that did crossover to an in-person visit, 73.1% did so because the telehealth visit was inadequate. Interestingly, the majority of these conversions did not occur due to insufficient physical examinations but rather because radiographic images could not be reviewed remotely. This suggests that technology failures, rather than an inability to accurately assess the condition of the patient, were a primary motivator for conversions to in-person visits. Unfortunately, more specifics regarding other types of technological failures during the telemedicine visits were not consistently recorded.

Our experience with the telehealth neurological exam has been similar to those of other institutions and our conversion to in-person visits seems to be even lower than previously reported series [25-29]. Although a larger number of spine patients experienced telemedicine failures in our cohort, this likely represents selection bias as more spine attendings participated in the study than cranial.

It is our hope that with the widespread adoption of telemedicine, these exam techniques will be further refined and validated thus further reducing the need for in-person visits during time such as this when social distancing is of utmost importance. While much of the material contained in this manuscript is not entirely novel, it attempts to think through many of the problems which will be commonly encountered by the neurosurgeon in the course of conducting a telemedicine exam, thereby mitigating future frustration and duplication of effort. Regardless of what the future holds, neurosurgical telemedicine is here, and it is our responsibility to consider how the focused neurosurgical exam can be adapted to overcome the shortcomings of a virtual encounter.

Limitations

As mentioned previously, we must also recognize potential shortcomings of the telemedicine examination. As such, there are certain components of the exam which are not amenable to telemedicine. For those patients where these diagnostic tests are of critical importance, in-person visits will be required. In our cohort, we had 19 instances in which the attending neurosurgeon felt that a more detailed evaluation was required prior to making a definitive surgical plan. Interestingly, we found that only one of these conversions was due to the need for a more detailed motor exam. Although anecdotal, this appears to demonstrate that strength examinations can accurately be performed for the majority of patients during a telemedicine visit. However, we recognize that the exact details of the motor and reflex exams were not consistently reported within our cohort. Therefore, the accuracy of our proposed tests cannot be truly assessed. Furthermore, the majority of consultations did not discuss technological failures, such as the inability to utilize the video application, so conversions to telephone usage only cannot be accurately evaluated in this study.

## Conclusions

This study outlines a number of adaptations to the traditional neurosurgical exam to account for the challenges of the telemedicine encounter. While these techniques require validation and discussion within the neurosurgical community, our techniques and diagrams should help in the early implementation of telemedicine programs. Our described techniques will hopefully serve as a springboard for further prospective analyses.

## References

[1] Fraser HS, McGrath SJ (2000). Information technology and telemedicine in sub-Saharan Africa. BMJ.

[2] Higgins CA, Conrath DW, Dunn EV (1984). Provider acceptance of telemedicine systems in remote areas of Ontario. J Fam Pract.

[3] Casey M, Hayes PS, Heaney D (2013). Implementing transnational telemedicine solutions: a connected health project in rural and remote areas of six Northern Periphery countries series on European collaborative projects. Eur J Gen Pract.

[4] Basil GW, Eichberg DG, Perez-Dickens M (2020). Letter: implementation of a neurosurgery telehealth program amid the COVID-19 crisis-challenges, lessons learned, and a way forward. Neurosurgery.

[5] Eichberg DG, Basil GW, Di L (2020). Telemedicine in neurosurgery: lessons learned from a systematic review of the literature for the COVID-19 Era and beyond. Neurosurgery.

[6] Eichberg DG, Shah AH, Luther EM (2020). Letter: academic neurosurgery department response to COVID-19 pandemic: the University of Miami/Jackson Memorial Hospital model. Neurosurgery.

[7] Luther E, Burks J, Eichberg DG (2021). Neuro-oncology practice guidelines from a high-volume surgeon at the COVID-19 epicenter. J Clin Neurosci.

[8] Eichberg DG, Epstein RH, Dexter F (2020). Building a brain tumor practice: objective analysis of referral patterns and implications for the growth of a subspecialty surgical program. Cureus.

[9] Burks JD, Luther EM, Govindarajan V, Shah AH, Levi AD, Komotar RJ (2020). Early changes to neurosurgery resident training during the COVID-19 pandemic at a large U.S. academic medical center. World Neurosurg.

[10] Kahn EN, La Marca F, Mazzola CA (2016). Neurosurgery and telemedicine in the United States: assessment of the risks and opportunities. World Neurosurg.

[11] Handschu R, Littmann R, Reulbach U (2003). Telemedicine in emergency evaluation of acute stroke: interrater agreement in remote video examination with a novel multimedia system. Stroke.

[12] Craig J, McConville J, Patterson V, Wootton R (1999). Neurological examination is possible using telemedicine. J Telemed Telecare.

[13] Yager PH, Clark ME, Dapul HR, Murphy S, Zheng H, Noviski N (2014). Reliability of circulatory and neurologic examination by telemedicine in a pediatric intensive care unit. J Pediatrics.

[14] Dick J, Guiloff R, Stewart A (1984). Mini-mental state examination in neurological patients. J Neurol Neurosurg Psychiatry.

[15] Cook C, Roman M, Stewart KM, Leithe LG, Isaacs R (2009). Reliability and diagnostic accuracy of clinical special tests for myelopathy in patients seen for cervical dysfunction. J Orthop Sports Phys Ther.

[16] Sohrab SA, Gelb D (2016). Value of self-induced plantar reflex in distinguishing Babinski from withdrawal. Neurology.

[17] House JW, Brackmann DE (1985). Facial nerve grading system. Otolaryngol Head Neck Surg.

[18] Choi JM, Lee HB, Park CS, Oh SH, Park KS (2007). PC-based tele-audiometry. Telemed e-Health.

[19] Swanepoel DW, Mngemane S, Molemong S, Mkwanazi H, Tutshini S (2010). Hearing assessment—reliability, accuracy, and efficiency of automated audiometry. Telemed e-Health.

[20] Bagai A, Thavendiranathan P, Detsky AS (2006). Does this patient have hearing impairment?. JAMA.

[21] Thomas KE, Hasbun R, Jekel J, Quagliarello VJ (2002). The diagnostic accuracy of Kernig's sign, Brudzinski's sign, and nuchal rigidity in adults with suspected meningitis. Clin Infect Dis.

[22] Waghdhare S, Kalantri A, Joshi R, Kalantri S (2010). Accuracy of physical signs for detecting meningitis: a hospital-based diagnostic accuracy study. Clin Neurol Neurosurg.

[23] Fortin JD, Falco FJ (1997). The Fortin finger test: an indicator of sacroiliac pain. Am J Orthop (Belle Mead NJ).

[24] Cattley P, Winyard J, Trevaskis J, Eaton S (2002). Validity and reliability of clinical tests for the sacroiliac joint. A review of literature. Australas Chiropr Osteopathy.

[25] Laslett M, Aprill CN, McDonald B, Young SB (2005). Diagnosis of sacroiliac joint pain: validity of individual provocation tests and composites of tests. Man Ther.

[26] Kolcun JPG, Ryu WHA, Traynelis VC (2020). Systematic review of telemedicine in spine surgery. J Neurosurg Spine.

[27] Blue R, Yang AI, Zhou C (2020). Telemedicine in the era of coronavirus disease 2019 (COVID- 19): a neurosurgical perspective. World Neurosurg.

[28] Daggubati LC, Eichberg DG, Ivan ME (2020). Telemedicine for outpatient neurosurgical oncology care: lessons learned for the future during the COVID-19 pandemic. World Neurosurg.

[29] Mouchtouris N, Lavergne P, Montenegro TS (2020). Telemedicine in neurosurgery: lessons learned and transformation of care during the COVID-19 pandemic. World Neurosurg.

